# Radiation therapy patients’ interest in psychedelic-assisted therapy: results of a survey

**DOI:** 10.1186/s13014-025-02686-9

**Published:** 2025-07-21

**Authors:** Jino Park, Rufus Banks, Akul Munjal, Krishna Hanubal, Nicholas Peterson, Garrett Harada, Michael A. Hoyt, Erin H. Healy, Jeremy P. Harris

**Affiliations:** 1 Department of Radiation Oncology, University of California Irvine, Orange, CA USA; 2 University of California Irvine School of Medicine, Irvine, CA USA; 3 Department of Population Health and Disease Prevention, University of California Irvine, Ivrine, CA USA

**Keywords:** Psychedelic-assisted therapy, Radiation therapy, Cancer-related anxiety, Depression, Mental health symptoms, Palliative care

## Abstract

**Background:**

Comorbid mental health symptoms impact 30–40% of cancer patients, significantly compromising treatment adherence and increasing mortality rates. Among patients undergoing radiation therapy, which is delivered with palliative intent in nearly half of all cases and for those nearing end-of-life, these rates may be even higher. Emerging research underscores the promising potential of psychedelic-assisted therapy (PAT) in alleviating cancer-related psychological distress. However, the perspectives of cancer patients on the therapeutic utility of psychedelics remain unexplored.

**Methods and materials:**

Adult patients with a cancer diagnosis were recruited in Radiation Oncology Clinic between May 2023 and August 2024. They included patients being evaluated before, during, or after radiation therapy. Data on demographics, medical history, prior psychedelic use, and measures of mental health burden and quality of life using validated questionnaires were collected to assess interest in PAT and factors associated with such interest.

**Results:**

100 patients enrolled in the study. 43% expressed interest in PAT, while 31% were opposed, and 26% were unsure. Prior diagnoses of mental health disorders like anxiety and depression, prior recreational psychedelic use, younger age, and male sex were positively associated with interest in PAT. Notably, patients with higher levels of depression, worse spiritual well-being, worse demoralization, worse quality of life, and more pain, symptoms that are targeted with PAT, were more likely to be receptive to it. Hesitancy was primarily attributed to a lack of information, cited by 43% of those not interested or unsure.

**Conclusion:**

Psychedelic-assisted therapy represents a promising avenue to address critical gaps in cancer-related mental health care, and this study suggests that a substantial portion of cancer patients are receptive to and curious about this approach. The primary barrier to acceptance is informational, emphasizing the need for further research and education to dispel misconceptions and increase awareness of the safety and efficacy of psychedelic therapies. Future work should explore provider perspectives, patient outcomes, and the integration of PAT into palliative care frameworks.

**Supplementary Information:**

The online version contains supplementary material available at 10.1186/s13014-025-02686-9.

## Background

Approximately 30–40% of cancer patients suffer from comorbid mood disorders such as anxiety and depression, with many developing such conditions after their cancer diagnosis [1, 2]. These mood symptoms not only are linked to lower treatment adherence and higher cancer mortality, but also incur a greater financial burden on the individual and health systems [3, 4]. Conventional management involves a combination of psychotherapy (e.g. cognitive-behavioral therapy) and medications (e.g. SSRIs), but these strategies are limited by delayed onset of action and lack robust evidence of effectiveness, particularly in the cancer population [5, 6]. Additionally, separately described cancer-related symptoms such as existential distress and demoralization—conditions which do not meet criteria for DSM-5 diagnoses and for which there are no specifically applicable medications—represent unmet needs that warrant novel therapeutic approaches to provide more comprehensive oncologic care and to combat the mental burden of a cancer diagnosis. [7]

An approach that has been recently tested for depression and anxiety symptoms in the cancer population is psychedelic-assisted psychotherapy (PAT). Research into clinical applications of psychedelic medications dates back to the 1960 s, and more recently emerging studies have demonstrated some benefits to LSD (lysergic acid diethylamide), psilocybin, ketamine, and MDMA (3,4-methylenedioxymethamphetamine) in treating treatment-resistant depression, suicidality, end-of-life anxiety, existential distress, PTSD (post-traumatic stress disorder), and substance use disorders [8–13]. For example, a double blinded, randomized placebo-controlled trial demonstrated that just two doses of ketamine administered 24 h apart achieved meaningful reduction in suicidal ideations in the short-term, while long-term follow up of patients who underwent single-dose psilocybin-assisted psychotherapy showed sustained reductions in depression, hopelessness, demoralization, and death anxiety in cancer patients up to 4.5 years later [8, 14]. A majority of the early clinical trials of PAT have centered around patients with terminal cancer. Within radiation oncology, 40–50% of treatments are given with palliative intent, and so there is significant overlap in the population who also have cancer-related mental health symptoms for which PAT may have a role. [15]

Despite growing evidence supporting the therapeutic use of psychedelic medications, they remain classified as Schedule I drugs, meaning they are considered to have no accepted medical use and a high potential for abuse. While it is thought that the legal classification, underground market, historical context, and anecdotes of negative experiences with psychedelics likely contribute to stigma and hesitancy around their clinical investigation and implementation, the opinions of cancer patients are understudied. To assess patient interest in PAT for treating cancer-related comorbid mental health symptoms, we performed a survey study of patients being treated with radiation therapy.

## Methods and materials

### Study participants

This single-institution prospective study recruited adult patients above age 18 who were undergoing, had recently completed, or were planning to start radiation therapy between May 2023 and August 2024. Participants were enrolled during on-treatment visits, follow-up appointments, and simulations for radiation planning. Eligibility required English language fluency. This study received Institutional Review Board approval.

### Survey design

An English-language survey was developed to collect demographic data, views on PAT, measures of mental health burden and health-related quality of life (HRQoL). The survey included questions about prior diagnoses of mood disorders and recreational psychedelic use, and additional medical history was obtained via electronic health records. To assess interest in PAT, participants were asked the following primary question: “Research is growing in the potential for psychedelics to help treat conditions like anxiety, depression, and existential suffering. If you have experienced any of these issues, would you be interested in treatment with psychedelic therapy?” Answer choices included “yes”, “no”, and “not sure”. Reasons for being against the use of PAT were recorded in multiple choice format from those who answered “no” or “not sure”. HRQoL was measured using the Hospital Anxiety and Depression Scale (HADS), Functional Assessment of Chronic Illness Therapy – Spiritual Well-Being (FACIT-Sp-12), Demoralization Scale (DS), EORTC Quality of Life Questionnaire (EORTC QLQ-C30), and Brief Pain Inventory (BPI). These surveys are previously validated surveys widely utilized within and outside of the field of oncology to screen or assess patients’ levels of mood symptoms, spiritual well-being, demoralization, functional and mental quality of life, and pain, respectively. HRQoL surveys were scored as previously described; higher scores of HADS, DS, EORTC QLQ-C30, and BPI and lower scores of FACIT-Sp-12 correlate with worse HRQoL. [16–20]

### Statistics

Descriptive statistics were used to gauge patients’ interest in psychedelic therapy and illustrate baseline characteristics including demographic data and medical history. To create a binary outcome, those who answered the primary question with “no” or “not sure” were grouped together and compared to those who answered “yes”. Associations of interest in PAT with baseline characteristics were evaluated with univariate linear regression. Associations of interest in PAT with HRQoL scores were evaluated using Mann–Whitney U tests. Significance testing was two-tailed with a threshold of 0.05. Statistical analyses were performed on R studio version 4.4.2 and Excel.

## Results

154 patients were screened for the survey, and 100 enrolled. Participants were informed that the study was voluntary and anonymous, and all provided consent and completed the full survey.

Of the 100 patients enrolled in the study, 63% were male and 37% female (Table 1). Median age was 61 (range 21–90). 29% of patients reported a prior diagnosis of mood disorders, most commonly anxiety (25%) and depression (17%). Primary cancer diagnoses were prostate 23%, breast 16%, head and neck 15%, skin 11%, CNS (central nervous system) 8%, gynecologic 8%, anorectal 7%, lung 5%, soft tissue sarcoma 4%, lymphoma 2%, and bladder 1%. 48% of patients were surveyed prior to receiving radiation therapy, 24% during radiation, and 28% after radiation. Radiation therapy intent was 76% definitive and 24% palliative. 25% of patients reported prior use of psychedelics, most commonly psilocybin (19%), LSD (15%), and MDMA (8%).

**Table 1:** Participants’ demographic data and medical history

Characteristics (n = 100)	Count (Percent)
Age (years)	61 median (21–90 range)
Female	37 (37%)
Male	63 (63%)
*Race*	
White	67 (67%)
Asian	13 (13%)
Black	3 (3%)
Other/Unknown	17 (17%)
*Education*	
Less than high school	4 (4%)
High school graduate	26 (26%)
Associate degree	7 (7%)
Bachelor’s degree	37 (37%)
Master’s degree	14 (14%)
Doctorate degree	12 (12%)
Prior diagnosis of mood disorder	29 (29%)
Anxiety	25 (25%)
Depression	17 (17%)
PTSD, bipolar disorder, or other	7 (7%)
History of psychedelic use	25 (25%)
Psilocybin	19 (19%)
LSD	15 (15%)
MDMA	8 (8%)
Ketamine	3 (3%)
DMT/ayahuasca	2 (2%)
Other	9 (9%)
*Primary Cancer*	
Prostate	23 (23%)
Breast	16 (16%)
Head and neck	15 (15%)
Skin	11 (11%)
CNS	8 (8%)
Gynecologic	8 (8%)
Anorectal	7 (7%)
Lung	5 (5%)
Sarcoma	4 (4%)
Lymphoma	2 (2%)
Bladder	1 (1%)
*Survey Timing*	
Before radiation	48 (48%)
During radiation	24 (24%)
After radiation	28 (28%)
*Radiation delivery intent*	
76 (76%)	
Definitive palliative	24 (24%)

**Fig. 1:**
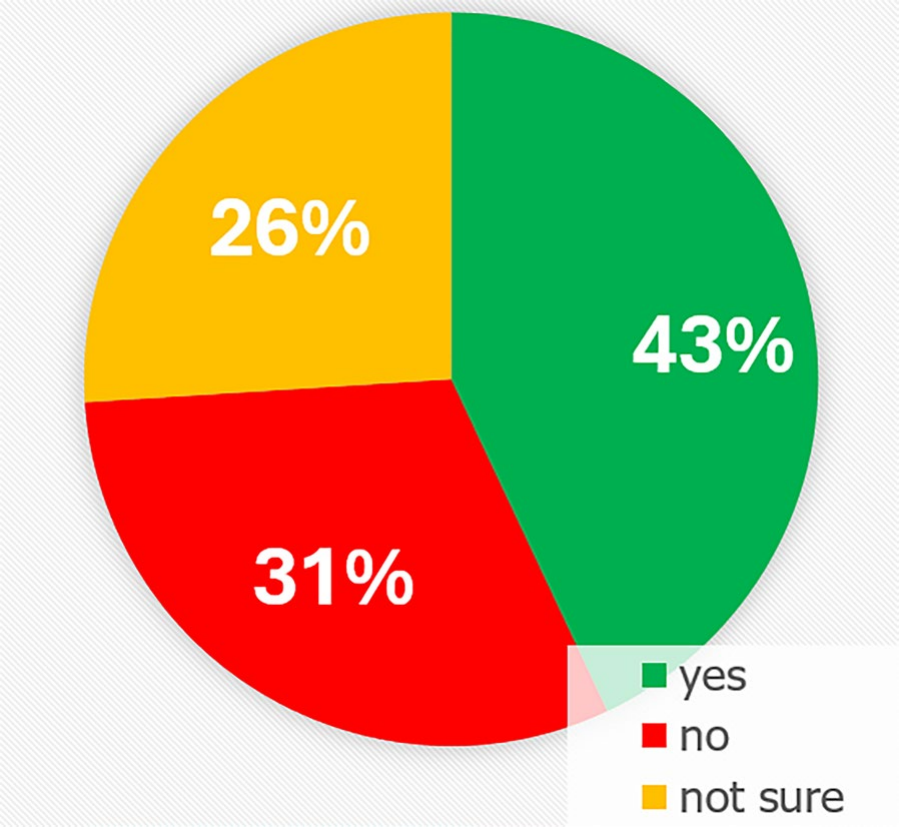
Pie chart demonstrating patients’ answers to the primary question: “Research is growing in the potential for psychedelics to help treat conditions like anxiety, depression, and existential suffering. If you have experienced any of these issues, would you be interested in treatment with psychedelic therapy?” (n = 100)

**Fig. 2:**
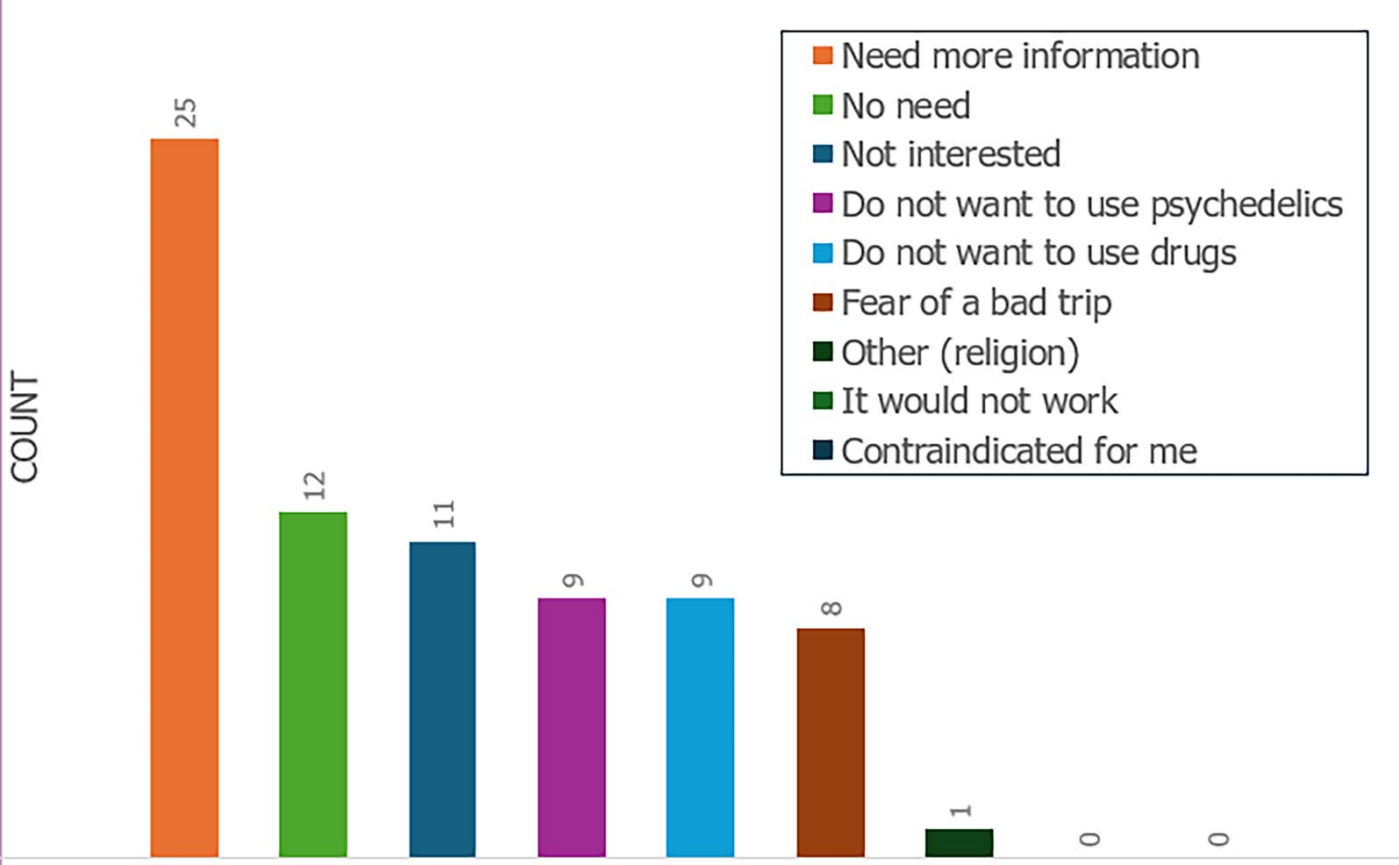
Bar graph illustrating the reasons for being against the use of psychedelics, among those who answered “no” or “not sure” (n = 57)

In response to the primary question, “[…] would you be interested in treatment with psychedelic therapy?” 43% of patients answered “yes”, 31% selected “no”, and 26% chose “not sure” (Fig. 1). The most common reason for answering “no” or “not sure” was “need more information” (43%, 25/57 patients). Other reasons included “no need” (21%), “not interested” (19%), “do not want to use psychedelics” (16%), “do not want to use drugs” (16%), “fear of a bad trip” (14%), and religion (2%) (Fig. 2).

**Fig. 3:**
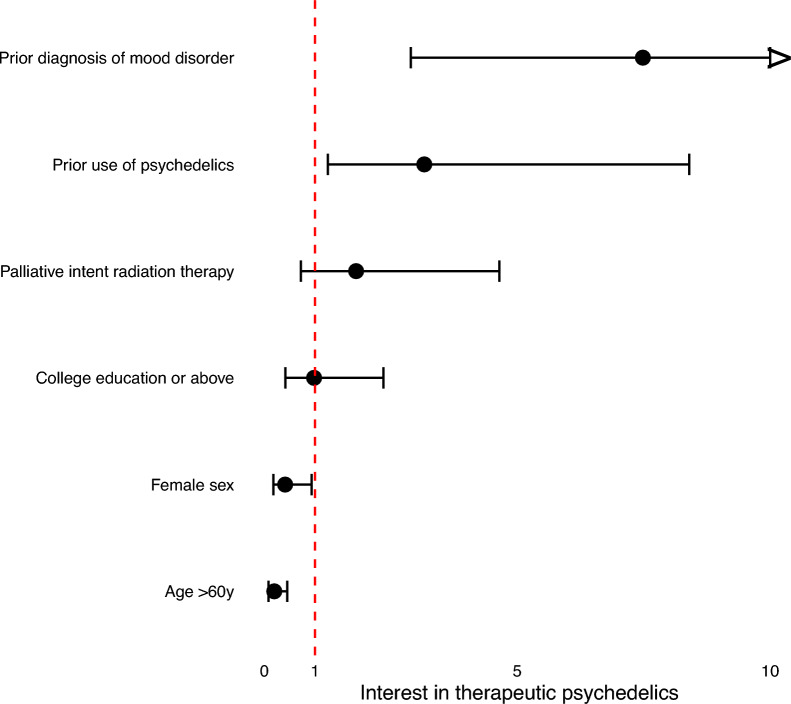
Forest plot of the odds ratios of various patient factors to psychedelic interest, based on univariate logistic regression

Interest in PAT was associated with a prior diagnosis of a mood disorder (OR 7.5, CI 2.9–21.5, *p* < 0.05), prior use of psychedelics (OR 3.2, CI 1.3–8.4, *p* < 0.05), male sex (female sex OR 0.4, CI 0.2–0.9, *p* < 0.05), and younger age (age > 60y OR 0.2, CI 0.1–0.4, *p* < 0.05). No correlation was found with radiation therapy intent (palliative intent OR 1.8, CI 0.7–4.6, *p* > 0.05) and education level (college or above OR 1.0, CI 0.4–2.4, *p* > 0.05) (Fig. 3).

**Fig. 4:**
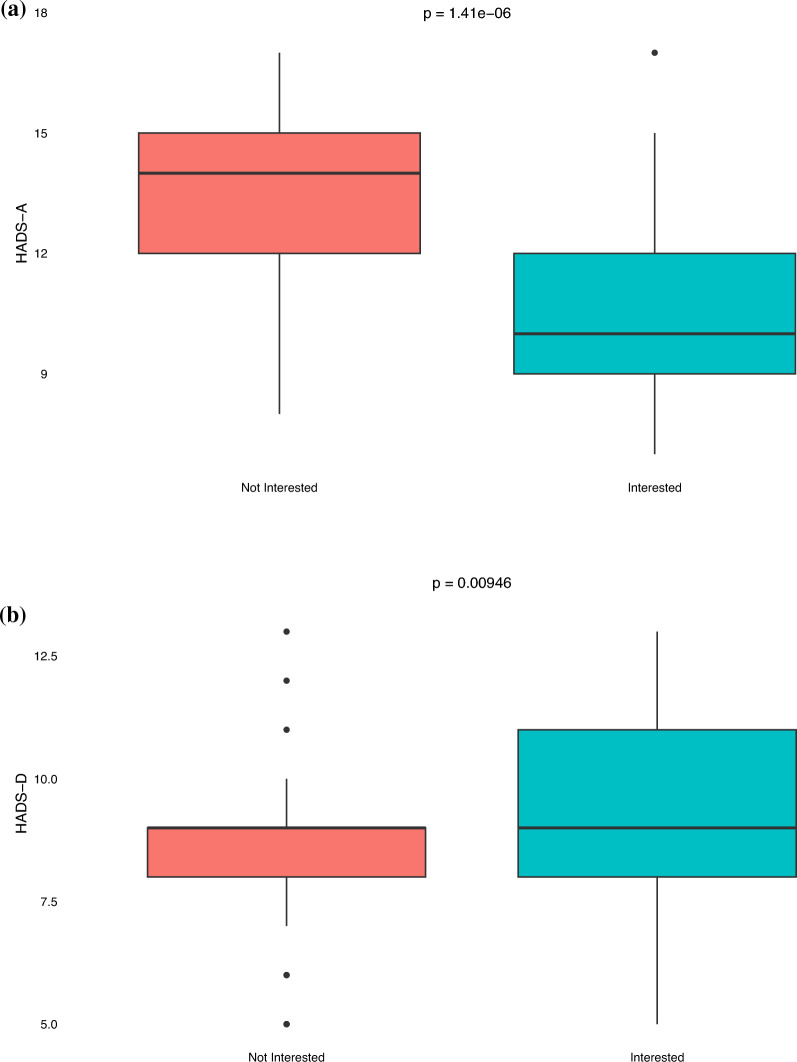

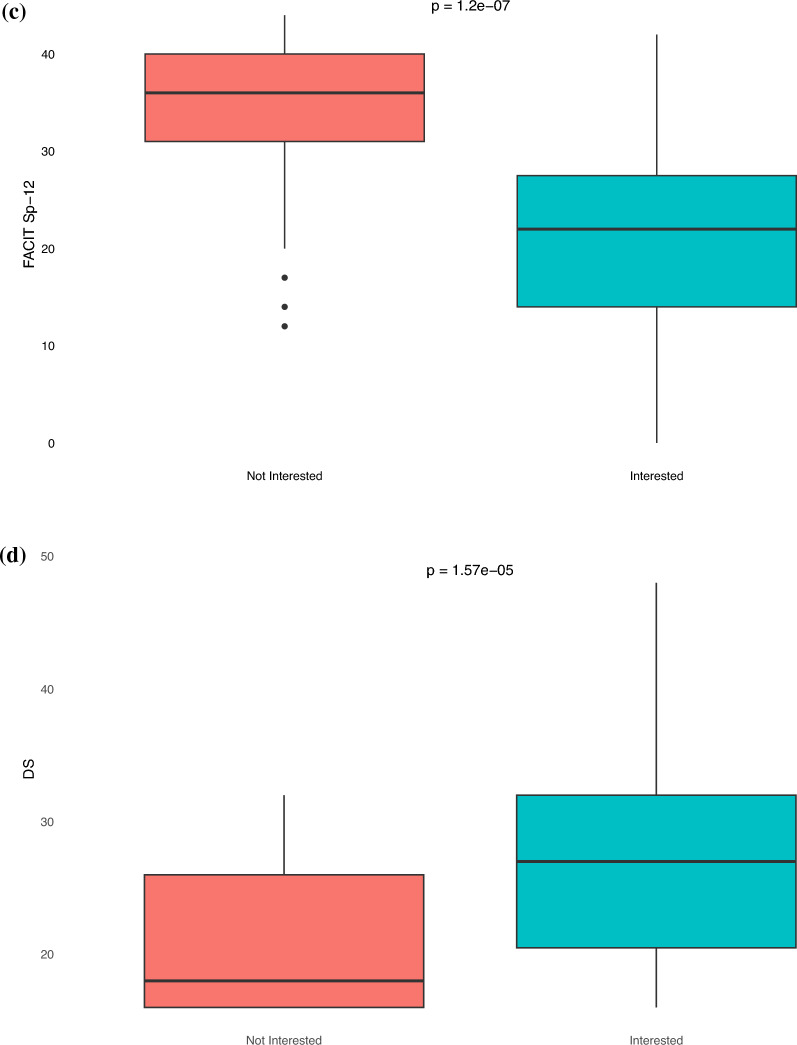

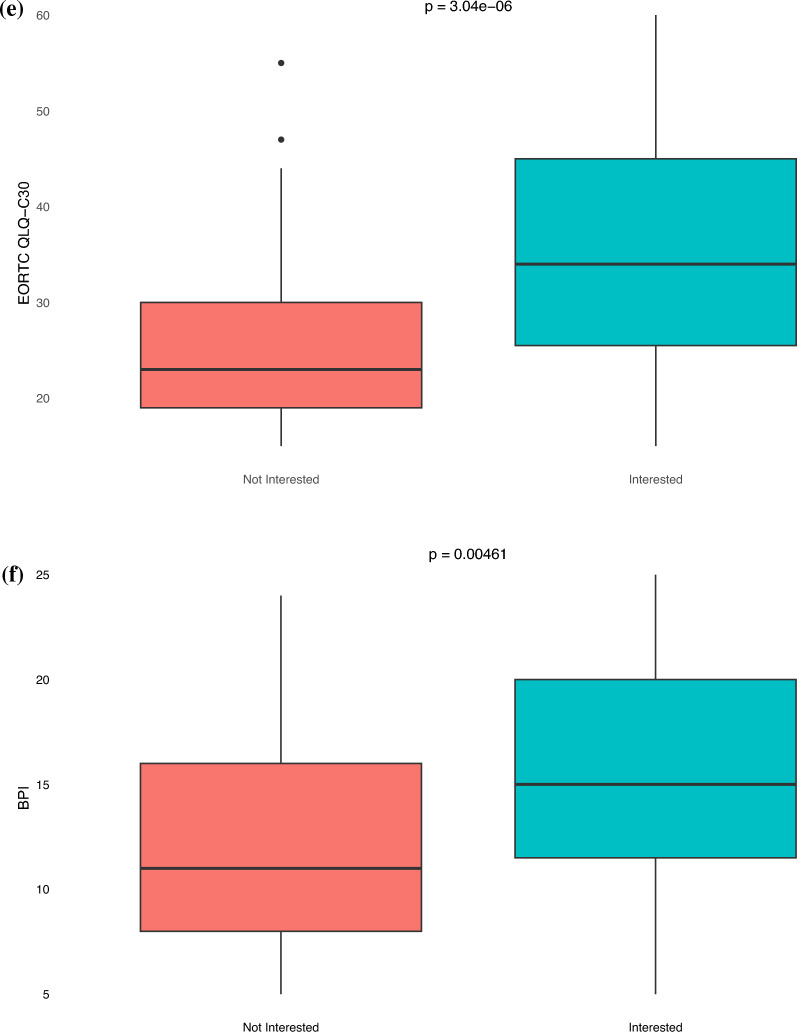
Box and whisker plots of HRQoL survey scores stratified by psychedelic interest. *p*-value are based on Mann–Whitney U test. **A** HADS-A (Hospital Anxiety and Depression Scale-Anxiety): Higher score indicates higher levels of anxiety. **B** HADS-D (Hospital Anxiety and Depression Scale-Depression): Higher score indicates higher levels of depression. **C** FACIT-Sp-12 (Functional Assessment of Chronic Illness Therapy – Spiritual Well-Being): Higher score indicates better spiritual well-being. **D** DS (Demoralization Scale): Higher score indicates worse demoralization. **E** EORTC QLQ-C30 (EORTC Quality of Life Questionnaire): Higher score indicates worse quality of life. **F** BPI (Brief Pain Inventory): Higher score indicates worse pain

Patients who expressed interest in PAT reported lower levels of anxiety compared to their counterpart (HADS-A median 10 vs 14, *p* < 0.05). In contrast, those interested in PAT had higher levels of depression compared to their counterpart (HADS-D median 9 vs 9, *p* < 0.05) (Fig. 4A and 4B). Patients interested in PAT had lower FACIT-Sp-12 scores, correlating to worse spiritual well-being, compared to those who were not interested (median 22 vs 36, *p* < 0.05) (Fig. 4C). Similarly, patients who expressed interested in PAT reported higher DS (median 27 vs 18, *p* < 0.05), EORTC QLQ-C30 (median 34 vs 23, *p* < 0.05), and BPI scores (median 15 vs 11, *p* < 0.05), correlating to worse demoralization, functional and mental quality of life, and pain, compared to those who were not interested (Figs. 4D-F).

## Discussion

This study explored the interest in psychedelic-assisted psychotherapy among cancer patients undergoing radiation therapy, revealing that nearly half of the respondents were open to such treatment for managing cancer-related mental health conditions. Notably, interest in PAT was higher among those with greater depressive symptoms, poorer spiritual well-being, higher demoralization, worse quality of life, and more pain—symptoms that are targeted with PAT.

Understanding the current status and potential applications of psychedelic medications in the United States requires examination of the historical context. In the mid-twentieth century, psychedelics were initially investigated for their therapeutic benefits in psychiatry, with particular interest in their ability to address mental health conditions such as depression, anxiety, PTSD, and addiction [21–23]. During this period, these substances were frequently administered in clinical settings to evaluate their effects on mood, cognition, and behavior. However, as the 1960 s progressed, recreational use of psychedelics surged, particularly within countercultural movements, and substances like LSD, psilocybin, and mescaline became emblematic of the broader anti-establishment sentiments [24]. As their popularity grew, so did public concerns about the potential risks of these substances, including their safety profiles and their capacity for abuse. In response to these concerns, the Controlled Substances Act of 1970 was enacted, which categorized substances into five schedules based on their potential for abuse, recognized medical use, and relative safety. Psychedelic substances became classified as Schedule I drugs. Some argue that this classification was influenced more by the political and social climate of the time marked by a growing"War on Drugs"narrative and fear of the counterculture rather than by definitive scientific evidence. At the time, ongoing research had already suggested potential therapeutic uses for psychedelics, but these findings were overshadowed by the political context. [25]

Following their Schedule I classification, psychedelic research virtually ceased for several decades. However, at the turn of the century, a resurgence of interest in the therapeutic potential of psychedelics emerged. Recent clinical trials have demonstrated promising results for the use of psychedelics in treating mental health symptoms, including several small randomized controlled trials demonstrating that even a single high-dose psilocybin session significantly reduced depression and anxiety by in patients with life-threatening cancer in about 80% of patients at 6-month follow up [8, 10, 11]. Both the scientific community and public interest have recognized this movement, as demonstrated by the FDA’s issuance of a draft guidance for psychedelic clinical investigations in June 2023. [26]

There are limited data into the patient perspective of psychedelic medications. In a survey of athletes, 61.2% expressed willingness to engage in PAT for persisting post-concussion symptoms, and among cancer patients in New Zealand, 59% were open to considering PAT for psychological distress [27, 28]. The results of our study support that sentiments towards therapeutic use of psychedelic medications may be shifting, with nearly half of patients treated with radiation therapy expressing some interest in psychedelic therapy to combat cancer-related mental health conditions. Notably, the main reason for hesitancy towards psychedelics is the lack of information. Furthermore, those who reported higher levels of depression, worse spiritual well-being, worse demoralization, worse quality of life, and more pain, symptoms that are targeted for psychedelic-assisted therapy, were more likely to be receptive to it. Interestingly, individuals with higher levels of anxiety demonstrated a reduced interest in psychedelics. Potential explanations include that patients with generalized anxiety disorder may perceive PAT as less targeted for their condition, given the current focus of clinical trials on depression, PTSD, and existential distress. Additionally, heightened anxiety may predispose apprehension towards novel treatments.

Limitations of this study include the innate selection and framing biases of a voluntary survey study, small sample size, and heterogenous diagnoses and prognoses. Future directions will incorporate the perspectives of physicians and other providers toward PAT, sub-analysis by cancer type or prognosis, and impact on HRQoL or survival with administration of palliative psychedelic therapy. To aid in integrating psilocybin-assisted psychotherapy into healthcare systems, Dorval et al. propose specific recommendations of actionable steps emphasizing the need for regulatory reform, clinician education, dedicated treatment infrastructure, and public awareness initiatives to ensure equitable access in palliative care settings. [29]

## Conclusion

This study highlights a substantial interest in PAT among cancer patients undergoing radiation therapy, particularly among those facing significant mental and physical health burdens, including depression, demoralization, pain, and poor quality of life. The primary barrier to potential interest appears to be a lack of information, underscoring the need for education and outreach to address misconceptions and inform patients about the completed and ongoing studies of PAT. Our findings suggest that there are unmet symptom needs in the oncology population and that there is significant patient interest in the clinical applications of PAT. Further research in the safety, efficacy, and implementation of psychedelics for the oncology population would be welcomed by patients.

## Supplementary Information

Additional file 1.

## Data Availability

The datasets used and/or analysed during the current study are available from the corresponding author on reasonable request.

## Abbreviations

SSRI: Selective serotonin receptor inhibitor
DSM: Diagnostic and Statistical Manual of Mental Illnesses
PAT: Psychedelic-assisted psychotherapy
LSD: Lysergic acid diethylamide
MDMA: 3,4-Methylenedioxymethamphetamine
PTSD: Post-traumatic stress disorder
HRQoL: Mental health burden and health-related quality of life
HADS: Hospital Anxiety and Depression Scale
FACIT-Sp-12: Functional Assessment of Chronic Illness Therapy–Spiritual Well-Being
DS: Demoralization Scale
EORTC QLQ-C30: European Organization for Research and Treatment of Cancer Quality of Life Questionnaire
BPI: Brief Pain Inventory
