# Supplementary material for: Radiation therapy patients’ interest in psychedelic-assisted therapy: results of a survey
Source: Radiat Oncol. 2025 Jul 21;20:114. doi: 10.1186/s13014-025-02686-9 (PMC12281839; doi:10.1186/s13014-025-02686-9)
Supplement: Supplementary file 1 — Additional file 1. [file 13014_2025_2686_MOESM1_ESM.pdf]

Date: ______________________

Name: ____	Date of birth: (Month/Day/Year) ______________________
Sex	☐ Female<br>☐ Male
Race	☐ White<br>☐ Asian<br>☐ Other Pacific Islander or Native Hawaiian<br>☐ Black<br>☐ American Indian or Alaska Native
Do you consider yourself Latino/Hispanic ethnicity?	☐ Yes<br>☐ No
Highest education attained	☐ Some school but did not graduate high school<br>☐ High school graduate or certificate of equivalency<br>☐ Associate degree<br>☐ Bachelor’s degree<br>☐ Master’s degree<br>☐ Doctorate degree
Have you been diagnosed with, treated, or monitored for a mood disorder? depression, anxiety, PTSD	*(select all that apply)*<br>☐ No (none)<br>☐ Depression<br>☐ Anxiety<br>☐ Post-traumatic stress disorder (PTSD)<br>☐ Other: ________________________________
Research is growing in the potential for psychedelics to help treat conditions like anxiety, depression, and existential suffering. If you have experienced any of these issues, would you be interested in treatment with psychedelic therapy?	☐ Yes<br>☐ No<br>☐ Not sure
If “no” or “not sure”, why not?	*(select all that apply)*<br>☐ Not interested<br>☐ Need more information<br>☐ It would not work<br>☐ Do not want to use drugs<br>☐ Do not want to use psychedelics<br>☐ No need<br>☐ Contraindicated for me<br>☐ Fear of a bad trip<br>☐ Other: ______________________________
Do you have any other concerns or objections to using psychedelics?	☐ No<br>☐ Yes: ______________________________

Have you ever used psychedelics in the past?	(select all that apply)<br>☐ No (none)<br>☐ LSD<br>☐ Psilocybin/magic mushrooms<br>☐ MDMA<br>☐ Ketamine<br>☐ Mescaline/peyote<br>☐ DMT/ayahuasca<br>☐ Salvia<br>☐ MDA/sassafras<br>☐ Other: ______________________________

Read each item below and mark the reply which comes closest to how you have been feeling in the **past week.**

	Very Poor						Excellent
How would you rate your overall health during the past week?	1	2	3	4	5	6	7
	Very Poor						Excellent
How would you rate your overall quality of life during the past week?	1	2	3	4	5	6	7

I feel tense or 'wound up'	Not at all	From time to time, occasionally	A lot of the time	Most of the time
I still enjoy the things I used to enjoy	Definitely as much	Not quite so much	Only a little	Hardly at all
I get a sort of frightened feeling as if something awful is about to happen	Not at all	A little, but it doesn't worry me	Yes, but not too badly	Very definitely and quite badly
I can laugh and see the funny side of things	As much as I always could	Not quite so much now	Definitely not so much now	Not at all
Worrying thoughts go through my mind	Only occasionally	From time to time but not too often	A lot of the time	A great deal of the time
I feel cheerful	Most of the time	Sometimes	Not often	Not at all
I can sit at ease and feel relaxed	Definitely	Usually	Not often	Not at all
I feel as if I am slowed down	Not at all	Sometimes	Very often	Nearly all the time
I get a sort of frightened feeling like 'butterflies' in the stomach	Not at all	Occasionally	Quite often	Very often
I have lost interest in my appearance	I take just as much care as ever	I may not take quite as much care	I don't take so much care as I should	Definitely
I feel restless as if I have to be on the move	Not at all	Not very much	Quite a lot	Very much indeed
I look forward with enjoyment to things	As much as ever I did	Rather less than I used to	Definitely less than I used to	Hardly at all
I get sudden feelings of panic	Not at all	Not very often	Quite often	Very often indeed
I can enjoy a good book or radio or TV program	Often	Sometimes	Not Often	Very seldom

	Not at all	A little bit	Somewhat	Quite a bit	Very much
I feel peaceful	☐	☐	☐	☐	☐
I have a reason for living	☐	☐	☐	☐	☐
My life has been productive	☐	☐	☐	☐	☐
I have trouble feeling peace of mind	☐	☐	☐	☐	☐
I feel a sense of purpose in my life	☐	☐	☐	☐	☐
I am able to reach down deep into myself for comfort	☐	☐	☐	☐	☐
I feel a sense of harmony within myself	☐	☐	☐	☐	☐
My life lacks meaning and purpose	☐	☐	☐	☐	☐
I find comfort in my faith or spiritual beliefs	☐	☐	☐	☐	☐
I find strength in my faith or spiritual beliefs	☐	☐	☐	☐	☐
My illness has strengthened my faith or spiritual beliefs	☐	☐	☐	☐	☐
I know that whatever happens with my illness, things will be okay	☐	☐	☐	☐	☐

	Not at All	A Little	Quite a Bit	Very Much
Do you have any trouble doing strenuous activities, like carrying a heavy shopping bag or a suitcase?	☐	☐	☐	☐
Do you have any trouble taking a long walk?	☐	☐	☐	☐
Do you have any trouble taking a short walk outside of the house?	☐	☐	☐	☐
Do you need to stay in bed or a chair during the day?	☐	☐	☐	☐
Do you need help with eating, dressing, washing yourself or using the toilet?	☐	☐	☐	☐
Were you limited in doing either your work or other daily activities?	☐	☐	☐	☐
Were you limited in pursuing your hobbies or other leisure time activities?	☐	☐	☐	☐
Did you feel tense?	☐	☐	☐	☐
Did you worry?	☐	☐	☐	☐
Did you feel irritable?	☐	☐	☐	☐
Did you feel depressed?	☐	☐	☐	☐
Have you had difficulty in concentrating on things, like reading a newspaper or watching television?	☐	☐	☐	☐
Have you had difficulty remembering things?	☐	☐	☐	☐
Has your physical condition or medical treatment interfered with your family life?	☐	☐	☐	☐
Has your physical condition or medical treatment interfered with your social activities?	☐	☐	☐	☐

How OFTEN did you have PAIN?	Never	Rarely	Occasionally	Frequently	Almost Constantly
What was the SEVERITY of your PAIN at its WORST?	None	Mild	Moderate	Severe	Very Severe
How much did PAIN INTERFERE with your usual or daily activities?	Not at all	A little bit	Somewhat	Quite a bit	Very much
What was the SEVERITY of your FATIGUE, TIREDNESS, OR LACK OF ENERGY at its WORST?	None	Mild	Moderate	Severe	Very Severe
How much did FATIGUE, TIREDNESS, OR LACK OF ENERGY INTERFERE with your usual or daily activities?	Not at all	A little bit	Somewhat	Quite a bit	Very much

Read each item below and circle the reply which comes closest to how you have been feeling in the **past 2 weeks.**

	Never	Sometimes	Often
There is little value in what I can offer others	☐	☐	☐
My life seems to be pointless	☐	☐	☐
My role in life has been lost	☐	☐	☐
I no longer feel emotionally in control	☐	☐	☐
No one can help me	☐	☐	☐
I feel that I cannot help myself	☐	☐	☐
I feel hopeless	☐	☐	☐
I feel irritable	☐	☐	☐
I do not cope well with life	☐	☐	☐
I have a lot of regret about my life	☐	☐	☐
I tend to feel hurt easily	☐	☐	☐
I feel distressed about what is happening to me	☐	☐	☐
I am not a worthwhile person	☐	☐	☐
I would rather not be alive	☐	☐	☐
I feel quite isolated or alone	☐	☐	☐
I feel trapped by what is happening to me	☐	☐	☐
